# Flavonoids from *Sideritis* Species: Human Monoamine Oxidase (hMAO) Inhibitory Activities, Molecular Docking Studies and Crystal Structure of Xanthomicrol

**DOI:** 10.3390/molecules20057454

**Published:** 2015-04-23

**Authors:** Fatma Pinar Turkmenoglu, İpek Baysal, Samiye Ciftci-Yabanoglu, Kemal Yelekci, Hamdi Temel, Salih Paşa, Nurten Ezer, İhsan Çalış, Gulberk Ucar

**Affiliations:** 1 Department of Pharmaceutical Botany, Faculty of Pharmacy, Hacettepe University, Ankara 06100, Turkey; E-Mail: nezer@hacettepe.edu.tr; 2 Department of Biochemistry, Faculty of Pharmacy, Hacettepe University, Ankara 06100, Turkey; E-Mails: ipekbaysal@hacettepe.edu.tr (I.B.); samiye@hacettepe.edu.tr (S.C-Y.); gulberk@hacettepe.edu.tr (G.U.); 3 Department of Bioinformatics and Genetics, Faculty of Engineering and Natural Sciences, Cibali Campus, Kadir Has University, Fatih, Istanbul 34083, Turkey; E-Mail: yelekci@khas.edu.tr; 4 Science and Technology Application and Research Center, Faculty of Pharmacy, Dicle University, Diyarbakir 21280, Turkey; E-Mail: htemel@dicle.edu.tr; 5 Department of Pharmaceutical Chemistry, Faculty of Pharmacy, Dicle University, Diyarbakir 21280, Turkey; E-Mail: spasa@dicle.edu.tr; 6 Department of Pharmacognosy, Faculty of Pharmacy, Near East University, Lefkoşa, Mersin-10, Turkey; E-Mail: ihsan.calis@neu.edu.tr

**Keywords:** *Sideritis*, flavonoid, xanthomicrol, salvigenin, monoamine oxidase, inhibition, molecular docking, X-ray diffraction investigation

## Abstract

The inhibitory effects of flavonoids on monoamine oxidases (MAOs) have attracted great interest since alterations in monoaminergic transmission are reported to be related to neurodegenerative diseases such as Parkinson’s and Alzheimer’s diseases and psychiatric disorders such as depression and anxiety, thus MAOs may be considered as targets for the treatment of these multi-factorial diseases. In the present study, four *Sideritis* flavonoids, xanthomicrol (**1**), isoscutellarein 7-*O*-[6'''-*O*-acetyl-β-d-allopyranosyl-(1→2)]-β-d-glucopyranoside (**2**), isoscutellarein 7-*O*-[6'''-*O*-acetyl-β-d-allopyranosyl-(1→2)]-6''-*O*-acetyl-β-d-glucopyranoside (**3**) and salvigenin (**4**) were docked computationally into the active site of the human monoamine oxidase isoforms (hMAO-A and hMAO-B) and were also investigated for their hMAO inhibitory potencies using recombinant hMAO isoenzymes. The flavonoids inhibited hMAO-A selectively and reversibly in a competitive mode. Salvigenin (**4**) was found to be the most potent hMAO-A inhibitor, while xanthomicrol (**1**) appeared as the most selective hMAO-A inhibitor. The computationally obtained results were in good agreement with the corresponding experimental values. In addition, the x-ray structure of xanthomicrol (**1**) has been shown. The current work warrants further preclinical studies to assess the potential of xanthomicrol (**1**) and salvigenin (**4**) as new selective and reversible hMAO-A inhibitors for the treatment of depression and anxiety.

## 1. Introduction

The genus *Sideritis* L. (Lamiaceae) comprises more than 150 species mainly distributed in countries of the Mediterranean-Macaronesian region [1]. *Sideritis* species , which have become very popular recently, are used as herbs either for the preparation of teas, or for their aromatic properties in local cuisines. They are found in a variety of shops, and marketed as mountain tea, dagçayi, adaçayi, yaylaçayi, malotira, té de Puerto, rabo de gato or zaharena in various Mediterranean countries [2,3,4]. Herbal teas prepared form the aerial parts of *Sideritis* species have also been used in traditional medicine for thousands of years in the treatment of gastrointestinal disorders such as stomach ache, indigestion and flatulence, to alleviate the symptoms of the common cold including fever, flu, sore throath, and bronchitis, and as analgesics, anticonvulsants, sedatives, tonics and diuretics [4,5,6,7]. Moreover, *Sideritis* species in the form of tea infusions are popularly used for improving memory function and cognitive ability [3]. The species of the genus are known to be rich in essential oils [4,8,9], diterpenes [4,10,11,12,13] and flavonoids [2,4,5,11,12,13,14,15,16].

Recently, the inhibitory effects of flavonoids on monoamine oxidases (MAO-A and MAO-B) have attracted great interest among researchers. Monoamine oxidases (MAOs) are flavoenzymes located in the outer membrane of the mitochondria that play an important role in the oxidative catabolism of endogenous and dietary amines [17,18]. MAO contains flavin adenine dinucleotide (FAD) as a co-factor and exists in two isoforms in mammals, named hMAO-A and hMAO-B [19]. MAO-A preferentially deaminates serotonin and norepinephrine, and is selectively inhibited by clorgyline, whereas MAO-B preferentially deaminates phenylethylamine and benzylamine and is selectively inhibited by L-deprenil [20,21]. Inhibitors of MAO-A are clinically used as antidepressants and anxiolytics [22,23], while MAO-B inhibitors are used in the treatment of Parkinson’s disease and in the management of symptoms associated with Alzheimer’s disease [24].

The studies on the crystal structures of MAOs and the interactions between MAOs and their ligands [24,25,26,27], have led to the design of new possible inhibitors by docking studies. Some currently used MAO inhibitors produce side effects due to a lack of selectivity towards one of the isoforms [28]. Thus, it is necessary to search for more potent, reversible and selective inhibitors of MAO-A and MAO-B. Despite the great amount of studies related to design and chemical synthesis of novel compounds as therapeutic agents or prodrugs, in recent decades identification of new compounds from natural sources or screening the novel biological activities of known natural compounds still attracts great interest.

A wide number of molecules of natural origin belonging to diverse chemical classes such as flavonoids, xanthones, proanthocyanidins, iridoids, alkaloids, curcumins, cannabinoids, *etc*. have been shown to inhibit human MAOs [29,30,31]. Among them, flavonoids have attracted more interest as they possess a variety of biological activities such as anti-oxidation and cardio-cerebrovascular protection besides their effects on the central nervous system. Their diverse activities give them extra advantages to be the potential multifunctional therapeutic agents for aging-related diseases [29,30]. Flavonoids are present in almost all terrestrial plants, and form part of the human diet via the consumption of vegetables, fruits, beverages and other plant-derived foods. The MAO inhibitory activities of some well-known flavonoids such as apigenin, kaempferol, quercetin, quercitrin, isoquercitrin and rutin have been previously demonstrated by *in vitro* and/or *in vivo* studies [29,30,31], however, few docking studies are available on these compounds in order to clarify the interaction of the compounds with the active site of hMAO [32].

In our previous studies, we have shown the anti-inflammatory, anti-nociceptive, anti-ulcerative antioxidant, aldose reductase and glutathione reductase activities of flavonoids isolated from various *Sideritis* species [6,16,33,34], in addition to the anti-inflammatory, anti-nociceptive, anti-ulcerative activities of their phenylethanoid glycosides and diterpenoids, and the anti-microbial activity of their iridoids and essential oils. Diuretic, anti-inflammatory, anti-spasmodic, antibacterial and antioxidant activities of different extracts obtained from several *Sideritis* species growing in Turkey were also previously demonstrated by our research group [6]. In the present work, hMAO-A and hMAO-B inhibitory activities of four *Sideritis* flavonoids, xanthomicrol (**1**), isoscutellarein 7-*O*-[6'''-*O*-acetyl-β-d-allopyranosyl-(1→2)]-β-d-glucopyranoside (**2**), isoscutellarein 7-*O*-[6'''-*O*-acetyl-β-d-allopyranosyl-(1→2)]-6''-*O*-acetyl-β-d-glucopyranoside (**3**), and salvigenin (**4**) were determined using recombinant human MAO. The molecular docking studies of these compounds were also performed to gain insights into the intermolecular interactions at the active site 3D space of both the hMAO-A and hMAO-B enzymes. In addition, the crystal structure of xanthomicrol (**1**), which was found to be the most selective hMAO-A inhibitor among those four flavonoids, has been described. The X-ray diffraction analysis demonstrates its exact structure with a dimeric molecule in the asymmetric unit.

## 2. Results and Discussion

### 2.1. Human Monoamine Oxidase (hMAO) Inhibitory Activities

**Figure 1:**
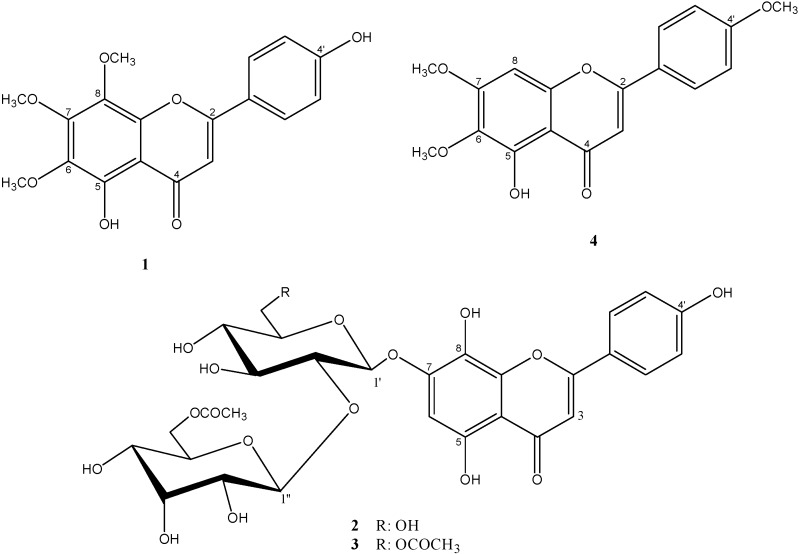
Structures of the four studied flavonoids: xanthomicrol (**1**), isoscutellarein 7-*O*-[6'''-*O*-acetyl-β-d-allopyranosyl-(1→2)]-β-d-glucopyranoside (**2**), isoscutellarein 7-*O*-[6'''-*O*-acetyl-β-d-allopyranosyl-(1→2)]-6''-*O*-acetyl-β-d-gluco-pyranoside (**3**) and salvigenin (**4**).

The hMAO inhibitory activities of the tested flavonoids (Figure 1) were investigated using commercially available recombinant hMAO-A and hMAO-B with tyramine as the substrate. Kinetic studies were performed in order to provide data for the interaction of four flavonoids with hMAO isoforms. The kinetic parameters K_m_ (Michaelis–Menten constant) and V_max_ (maximum rate) were determined by means of Lineweaver–Burk plots over a substrate range of 0.05–0.5 mM.

**Figure 2:**
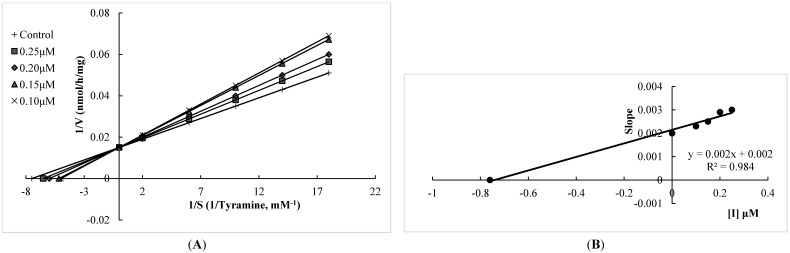
(**A**) Lineweaver–Burk plot of the competitive inhibition of hMAO-A by compound xanthomicrol (**1**) (**B**) Replot of data from the Lineweaver-Burk plot. The K_i_ value calculated from replots of the slopes of the Lineweaver-Burk plots *versus* inhibitor concentrations was found as 0.76 μM.

**Figure 3:**
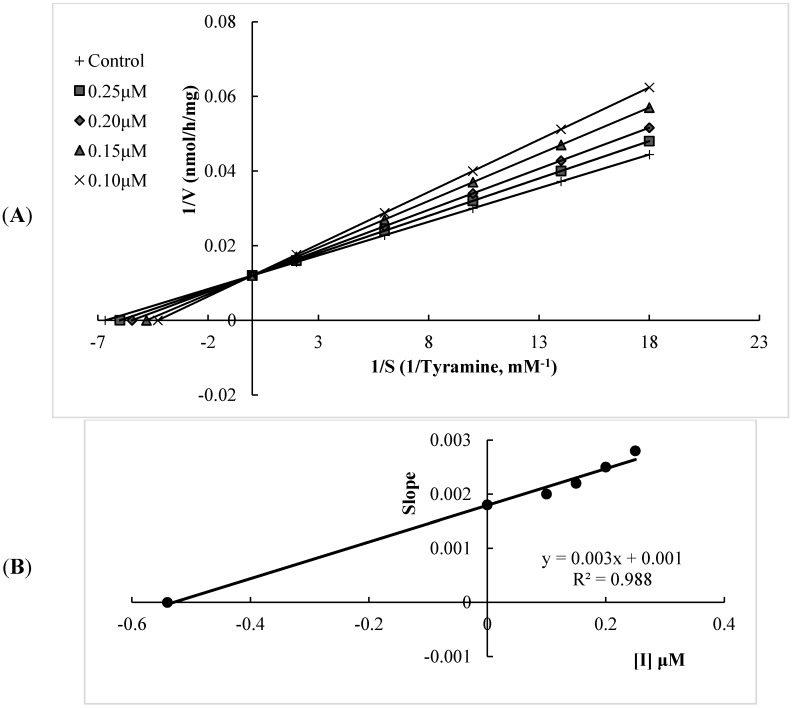
(**A**) Lineweaver-Burk plot of the competitive inhibition of hMAO-A by compound salvigenin (**4**); (**B**) Replot of data from the Lineweaver–Burk plot. The K_i_ value calculated from replots of the slopes of the Lineweaver–Burk plots *versus* inhibitor concentrations was found as 0.54 μM.

The MAO-A and MAO-B inhibitory potencies of the flavonoids and two well-known MAO-A and -B inhibitors (moclobemide and selegiline, respectively) are shown in Table 1. The results showed that all of the flavonoids tested possessed a degree of selectivity for hMAO-A, with experimental selectivity index (SI) values between 0.007 and 0.086, which suggests that these compounds are highly selective towards hMAO-A. Among them, xanthomicrol (**1**) appears as remarkably selective, with an experimental SI of 0.007 and selectivity was better than that of moclobemide, a highly potent and selective hMAO-A inhibitor (the experimental SI of moclobemide was determined as 0.010, Table 1). All of the flavonoids tested inhibited hMAO-A in a competitive mode. The kinetic behaviors of xanthomicrol (**1**) and salvigenin (**4**), which inhibited hMAO-A potently with experimental K_i_ values of 0.76 ± 0.03 and 0.54 ± 0.02 μM, respectively, are presented in Figure 2 and Figure 3. Salvigenin (**4**) appears as the most potent hMAO-A inhibitor among the tested compounds.

MAO inhibitors can be classified as reversible or irreversible, according to their interaction with hMAO isoforms. Although irreversible MAO inhibitors such as selegiline have been extensively used as clinical drugs, this type of inhibition is reported to induce serious cardiovascular toxic effects, mainly provoked by the inhibition of the peripheral MAO-A located in gut, liver and endothelium [35,36]. Besides, the slow and variable enzyme recovery following the withdrawal of irreversible inhibitors is a disadvantage in clinical use, since the turnover rate for MAO biosynthesis in the human brain seems to require about 40 days [37]. Competitive reversible inhibitors have been previously reported to have less influence in the enzyme recovery after withdrawal, and reversible MAO-A inhibitors, such as moclobemide, have no cardiovascular effects, since the ingested tyramine is able to displace the inhibitor from the MAO active site and be metabolized in the normal way by peripheral enzyme in gut and liver [35]. 

**Table 1:** Calculated and experimentally determined K_i_ values of the flavonoids, selegiline and moclobemide for hMAO isoforms A and B.

Compounds	ΔG for MAO-A	Calculated K_i_ for MAO-A(μM)	ΔG for MAO-B	Calculated K_i_ for MAO-B(μM)	Calculated SI **	Experimental K_i_ for MAO-A * (μM)	Experimental K_i_ for MAO-B * (μM)	Experimental SI **	Selectivity
**1**	−7.81	1.89	−5.78	64.25	0.029	0.76 ± 0.03	99.54 ± 7.44	0.007	MAO-A
**2**	−4.14	927.10	+5.78	-	-	1100.50 ± 99.20	75,600.00 ± 912.50	0.015	MAO-A
**3**	−3.79	1660.00	+8.92	-	-	2800.45 ± 180.50	80,500.00 ± 950.00	0.034	MAO-A
**4**	−8.27	0.867	−7.42	3.62	0.239	0.54 ± 0.02	6.27 ± 0.36	0.086	MAO-A
Selegiline						14.90 ± 1.33	0.36 ± 0.02	41.39	MAO-B
Moclobemide						0.014 ± 0.001	1.34 ± 0.03	0.010	MAO-A

* Each value represents the mean ± SEM of three experiments; ** The selectivity index (*SI*) was calculated as *K_i_* (MAO-A)/*K_i_* (MAO-B).

Thus, new selective and reversible inhibitors of MAO-A may be useful therapeutic agents for the treatment of psychiatric diseases and Alzheimer’s and Parkinson’s diseases devoid of undesirable side-effects, such as cheese effect. All of the flavonoids inhibited hMAO-A reversibly as shown in Table 2. Significant recovery of hMAO-A activity was observed after repeated washing of xanthomicrol (**1**) and salvigenin (**4**), indicating that these flavonoids are highly reversible inhibitors of hMAO isoforms.

**Table 2:** Reversibility of hMAO-A inhibition with flavonoids and moclobemide *.

Compounds (0.50 μM)	hMAO-A Inhibition (%)	
	Before Washing	After Washing
**1**	86.49 ± 5.21	9.23 ± 0.75
**2**	80.22 ± 5.66	10.90 ± 0.99
**3**	74.22 ± 4.89	13.35 ± 1.09
**4**	89.03 ± 5.02	8.66 ± 0.65
**Moclobemide**	91.22 ± 6.33	7.89 ± 0.52

* Each value represents the mean ± SEM of three experiment.

### 2.2. Molecular Docking Studies

**Figure 4:**
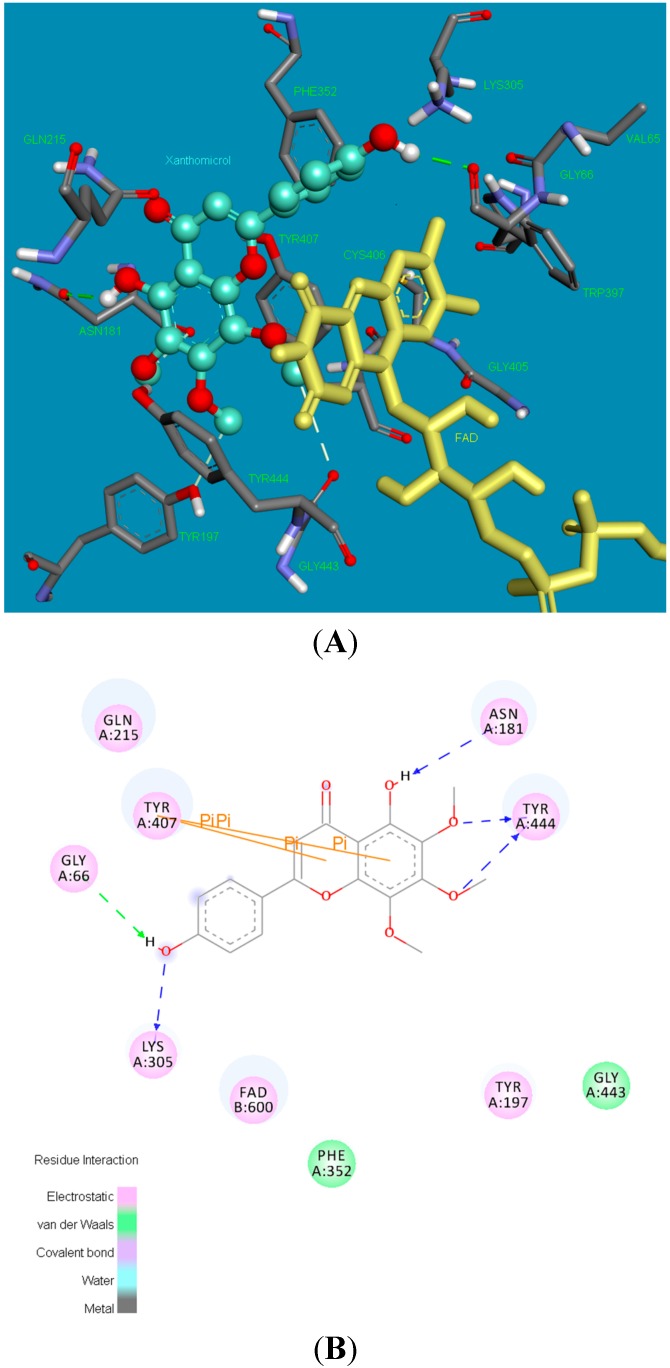
The 3 and 2 dimensional orientations of xanthomicrol (**1**) in the active site of hMAO-A. (**A**) 3D: Amino acid side chains are shown as sticks, the inhibitor is shown as a ball and stick (green), and the cofactor FAD is depicted as a yellow stick; (**B**) 2D: Purple color shows electrostatic and green color shows van der Waals atractions.

Figure 4 shows the binding pose (3D and 2D) of xanthomicrol (**1**) in the active site of hMAO-A isoform. The coumarine ring was inserted between the TYR444 and TYR407 residues, which constitute the hydrophobic cage of the active site, forms two π-π interactions with TYR407. The following five hydrogen bonds form between moieties of the xanthomicrol (**1**) and the side chains of the active site amino acids of the MAO-A isoform: between the -OH and ASN181, -OCH_3_ and TYR444 (two), -OH and LYS305 and -OH and GLY66.

**Figure 5:**
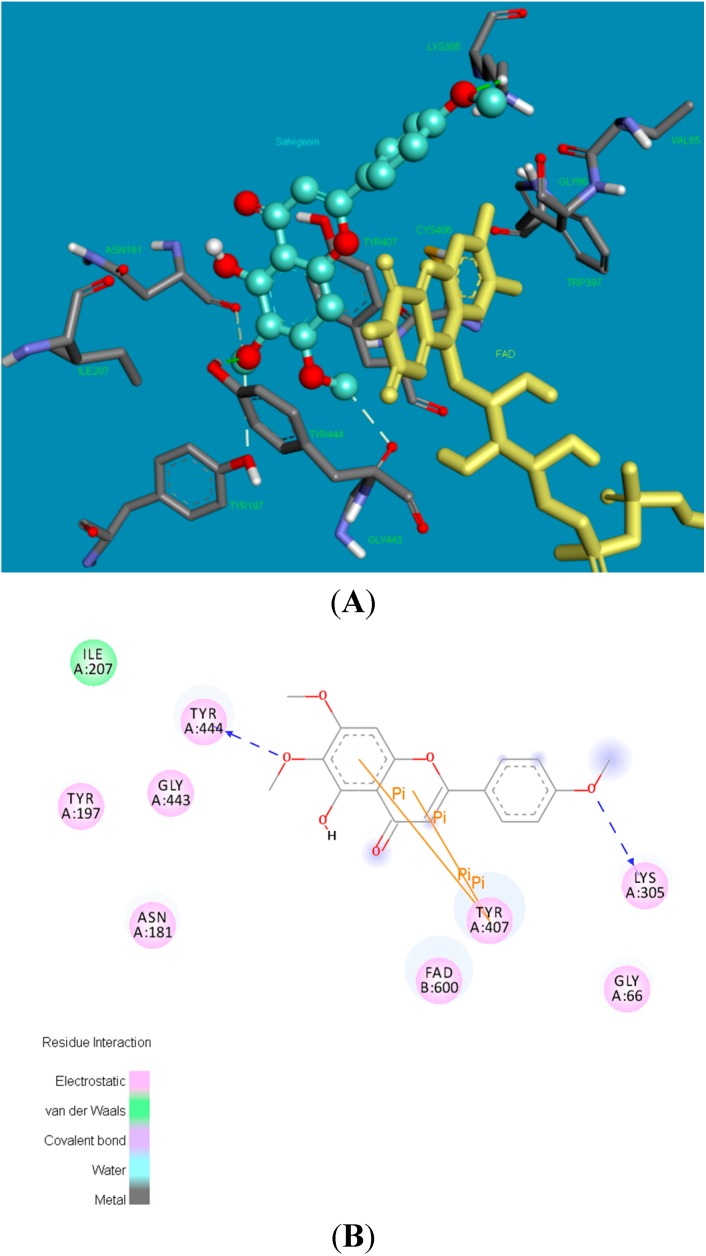
The 3 and 2 dimensional orientations of salvigenin (**4**) in the active site of hMAO-A. (**A**) 3D: Amino acid side chains are shown as sticks, the inhibitor is shown as a ball and stick (green), and the cofactor FAD is depicted as a yellow stick; (**B**) 2D: Purple color shows electrostatic and green color shows van der Waals atractions.

Analysis of the optimal binding mode for the salvigenin (**4**) in the hMAO-A active site cavity (Figure 5) revealed that this compound is docked in the vicinity of the FAD co-factor. Salvigenin forms various electrostatic and van der Waals interactions with the active site residues lining the cavity. A hydrogen bond occurs between the methoxy group of the salvigenin (**4**) and the hydroxy moiety of TYR444. The other strong electrostatic interactions occur between GLY443 backbone carbonyl and methoxy groups, and backbone carbonyl of ASN181 and methoxy group of the salvigenin (**4**). The benzene ring of the coumarine moiety of the salvigenin (**4**) was sandwiched between TYR444 and TYR407 making two π-π interactions.

**Figure 6:**
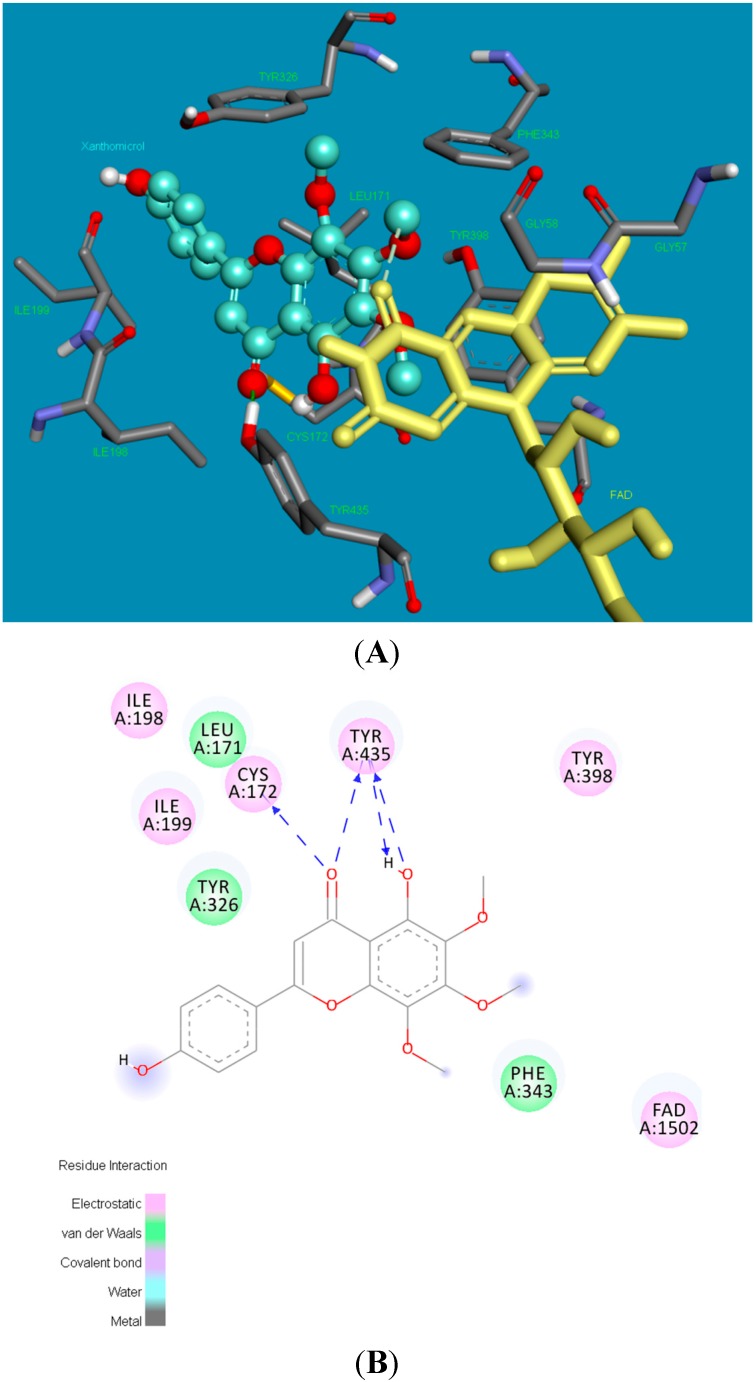
The 3 and 2 dimensional orientations of xanthomicrol (**1**) in the active site of hMAO-B. (**A**) 3D: Amino acid side chains are shown as sticks, the inhibitor is shown as a ball and stick (green), and the cofactor FAD is depicted as a yellow stick; (**B**) 2D: Purple color shows electrostatic and green color shows van der Waals atractions.

Figure 6 shows the poses of xanthomicrol (**1**) in the active side of hMAO-B in 3D and 2D depictions, respectively. Four hydrogen bonds form between the carbonyl and (2 bonds) and hydroxyl (2 bonds) of xanthomicrol (**1**) and side chains of CYS172 and TYR435. The other interacting side chain residues of hMAO-B with xanthomicrol (**1**) are ILE198, ILE199, CYS172, LEU171, TYR435, TYR398, TYR326 and PHE 343.

**Figure 7:**
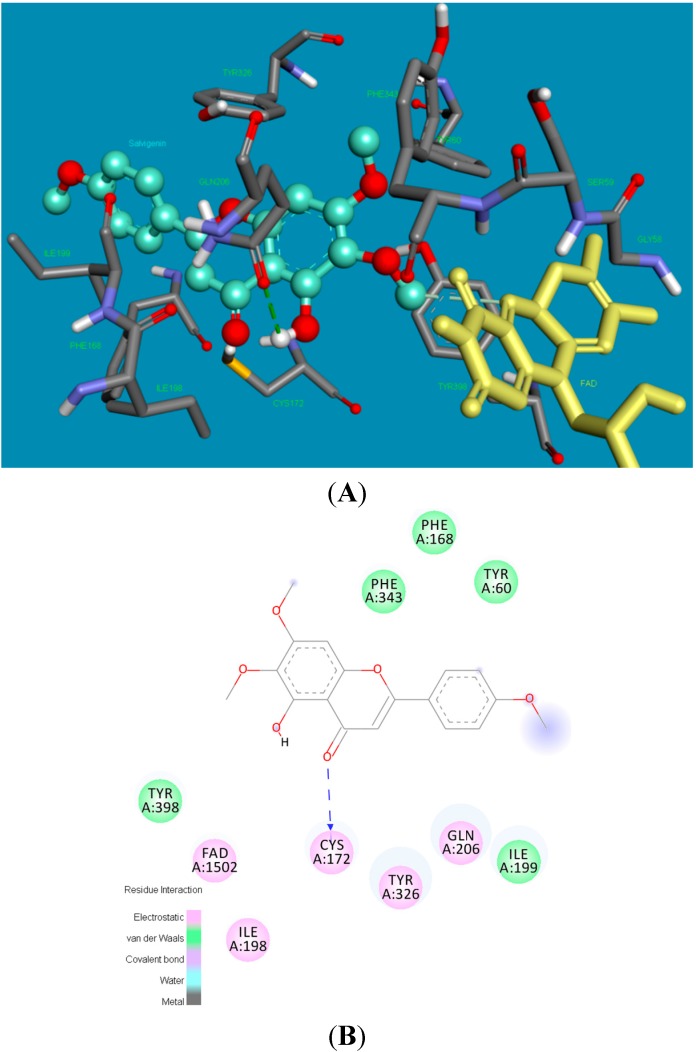
The 3 and 2 dimensional orientations of salvigenin (**4**) in the active site of hMAO-B. (**A**) 3D: Amino acid side chains are shown as sticks, the inhibitor is shown as a ball and stick (green), and the cofactor FAD is depicted as a yellow stick; (**B**) 2D: Purple color shows electrostatic and green color shows van der Waals atractions.

Figure 7 shows the binding pose of salvigenin (**4**) in the active site of hMAO-B isoform. The major interaction is the hydrogen bond that forms between the carbonyl group of coumarine ring and side chain residue of CYS172. The binding mode adopted by salvigenin (**4**) fits snugly within a cavity lined with hydrophobic amino acid residues. This hydrophobic pocket includes PHE168, TYR60, ILE199, TYR326, CYS172, ILE198, PHE343, GLN206 and TYR398 amino acids.

The potency and selectivity of xanthomicrol (**1**) and salvigenin (**4**) for the hMAO enzymes can be evaluated from the above data. The detailed analysis of Figure 5, Figure 7 and Table 1 suggest that salvigenin (**4**) is a better inhibitor of hMAO-A than of the MAO-B isoform. The reason for that the coumarine core is stabilized by more π–π interactions as well as other various attractions in the hMAO-A active side compared to the hMAO-B isozyme (calculated K_i_ = 0.867 μM for hMAO-A and calculated K_i_ = 1.89 μM for hMAO-B). However, xanthomicrol (**1**) is more selective towards hMAO-A than salvigenin (**4**) (calculated SIs of xanthomicrol (**1**) and salvigenin (**4**) were estimated as 0.029 and 0.239, respectively).

Inhibition of MAO by flavonoids appears to be dependent on the phenyl or hydroxyphenyl ring in the 2 position as well as a double bond in the 2 and 3 position of the structure. It has been previously demonstrated that flavonoids isolated from the whole plant and fruits of *Cayratia japonica* showed MAO inhibitory activity as follows: flavones > flavonols > flavone glycosides > flavanonols. In this series, apigenin inhibited mouse brain MAO-A with an IC_50_ value of 1.17 μM. It was suggested that MAO inhibitory activity decreased with increasing number of hydroxyl groups on the B ring of the flavones. The presence of electron donating hydrophilic hydroxyl groups in the *para* position of the B ring has also been reported to favour the MAO-A inhibition [30,31]. Thus, it can be postulated that the *p*-hydroxyl group in the B ring of xanthomicrol (**1**) increased the selectivity of this molecule towards hMAO-A.

Monoamine oxidases are responsible for the oxidative deamination of neurotransmitters and dietary amines. Of the two isoenzymes, MAO-A has attracted considerable attention because of its ability to affect the metabolism of serotonin [23]. Depletion of serotonin levels in the central nervous system (CNS) is suggested to be involved in the pathogenesis of depressive disorders [38]. It has been reported that MAO-A activity in the brain is elevated during depression, which causes depletion of serotonin in the brain [39]. Herbal medicines, such as flavonoids, are commonly used for the treatment of depression, along with conventional antidepressants [40]. Thus, in the present study we present four flavonoids isolated from *Sideritis* species exhibiting highly potent hMAO inhibitory activities. Among them, salvigenin (**4**) was found as the most potent hMAO-A activity, whereas xanthomicrol (**1**) appeared as the most selective one towards the hMAO-A isoform.

### 2.3. X-ray Crystallography

**Figure 8:**
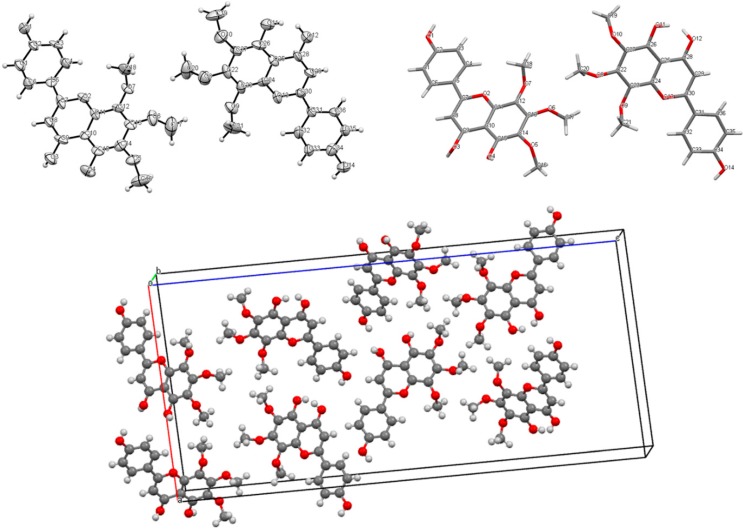
Crystal structure of xanthomicrol (**4**) showing two molecules in asymmetric unit and the crystal packing and alignment.

**Figure 9:**
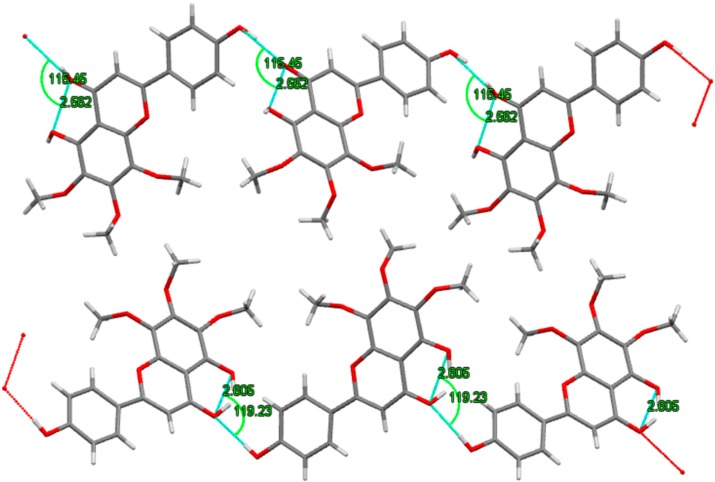
Hydrogen bond interactions of crystal.

The crystal structure of xanthomicrol (**4**) is shown in Figure 8. The bonding parameters are listed in Table 3. The structure of compound was solved by direct methods using SHELXS-97 [41]. Hydrogen atoms that bonded to carbon and oxygen were positioned geometrically. The asymmetric unit shows two molecules which stand together thanks to intermolecular hydrogen bonding (Figure 9). Both of hydrogen bonds can be seen in the crystal structure. The intramolecular hydrogen bond, which appears when donor and acceptor atoms are in the same molecule, has a length of 2.562 Å. On the other hand, the distance is 2.796 Å with 119.23° angle for intermolecular hydrogen bonding. Because of the geometrical constraints, it’s probably longer in intermolecular interactions [42].

The symmetry components can also be summarized such −x, −y, 1/2+z, 1/2−x, 1/2+y, 1/2+z and 1/2+x, 1/2−y, z correspond to symmetry operations; “2-fold screw axis with direction [0, 0, 1] at 0, 0, z with screw component [0, 0, 1/2]”, “Glide plane perpendicular to [1, 0, 0] with glide component [0, 1/2, 1/2]” and “lide plane perpendicular to [0, 1, 0] with glide component [1/2, 0, 0]”, respectively.

Although synthetic xanthomicrol (**1**) is commercially available (Apin Chemicals and BOC Sciences), to our knowledge, this is the first report on its crystal structure. The crystal structure of salvigenin (**4**) has already been reported [43].

**Table 3:** Bond angles (°) and bond distances (Å) for xanthomicrol (**1**).

Bond Angles (°)		Bond Angles (°)		Bond Angles (°)	
C6 C1 C2	121.3(10)	C14 C13 O6	120.7(11)	O9 C21 H21B	109.5
C6 C1 H1	119.3	C15 C14 O5	120.4(10)	H21A C21 H21B	109.5
C2 C1 H1	119.3	C15 C14 C13	120.6(11)	O9 C21 H21C	109.5
O1 C2 C3	122.5(9)	O5 C14 C13	118.9(11)	H21A C21 H21C	109.5
O1 C2 C1	117.5(10)	O4 C15 C14	122.1(10)	H21B C21 H21C	109.5
C3 C2 C1	119.7(10)	O4 C15 C10	118.9(10)	O8 C22 C27	120.8(10)
C2 C3 C4	120.8(10)	C14 C15 C10	118.9(10)	O8 C22 C23	118.6(10)
C2 C3 H3	119.6	O5 C16 H16A	109.5	C27 C22 C23	120.3(10)
C4 C3 H3	119.6	O5 C16 H16B	109.5	C24 C23 C22	120.0(10)
C5 C4 C3	118.6(11)	H16A C16 H16B	109.5	C24 C23 O9	119.8(10)
C5 C4 H4	120.7	O5 C16 H16C	109.5	C22 C23 O9	120.1(10)
C3 C4 H4	120.7	H16A C16 H16C	109.5	O13 C24 C23	118.6(10)
C4 C5 C6	121.5(11)	H16B C16 H16C	109.5	O13 C24 C25	120.1(11)
C4 C5 C7	120.2(10)	O6 C17 H17A	109.5	C23 C24 C25	121.2(11)
C6 C5 C7	118.3(10)	O6 C17 H17B	109.5	C24 C25 C26	117.7(10)
C1 C6 C5	118.0(10)	H17A C17 H17B	109.5	C24 C25 C28	118.2(11)
C1 C6 H6	121.0	O6 C17 H17C	109.5	C26 C25 C28	124.0(10)
C5 C6 H6	121.0	H17A C17 H17C	109.5	O11 C26 C27	119.4(10)
C8 C7 O2	121.5(11)	H17B C17 H17C	109.5	O11 C26 C25	120.2(10)
C8 C7 C5	128.1(11)	O7 C18 H18A	109.5	C27 C26 C25	120.4(9)
O2 C7 C5	110.3(9)	O7 C18 H18B	109.5	O10 C27 C26	121.9(9)
C7 C8 C9	121.3(11)	H18A C18 H18B	109.5	O10 C27 C22	117.8(10)
C7 C8 H8	119.4	O7 C18 H18C	109.5	C26 C27 C22	120.2(10)
C9 C8 H8	119.4	H18A C18 H18C	109.5	O12 C28 C29	122.7(10)
O3 C9 C8	122.3(12)	H18B C18 H18C	109.5	O12 C28 C25	119.3(11)
O3 C9 C10	120.1(11)	O10 C19 H19A	109.5	C28 C29 H29	119.0 (9)
C8 C9 C10	117.4(9)	O10 C19 H19B	109.5	C29 C28 C25	118.0(10)
C11 C10 C15	119.6(10)	H19A C19 H19B	109.5	C29 C30 O13	120.0(10)
C11 C10 C9	117.6(10)	O10 C19 H19C	109.5	C29 C30 C31	128.0(8)
C15 C10 C9	122.8(10)	H19A C19 H19C	109.5	C30 C29 C28	122.0(10)
C10 C11 O2	122.6(10)	H19B C19 H19C	109.5	C30 C29 H29	119.0
C10 C11 C12	121.7(10)	O8 C20 H20A	109.5	O13 C30 C31	111.8(10)
O2 C11 C12	115.6(9)	O8 C20 H20B	109.5	C36 C31 C32	114.9(9)
O7 C12 C13	122.0(10)	H20A C20 H20B	109.5	C36 C31 C30	122.4(10)
O7 C12 C11	120.5(10)	O8 C20 H20C	109.5	C32 C31 C30	122.5(10)
C13 C12 C11	117.2(9)	H20A C20 H20C	109.5	C33 C32 C31	122.3(10)
**Bond Distances (Å)**		**Bond Distances (Å)**		**Bond Distances (Å)**	
C1 H1	0.93 1	C15 O4	1.35(1) 1	C22 C27	1.39(2)
C1 C2	1.39 (1)	C16 H16A	0.96 1	C22 O8	1.34(1) 1
C1 C6	1.37(2)	C16 H16B	0.96 1	C23 C24	1.36(2)
C2 C3	1.36(2)	C16 H16C	0.96 1	C23 O9	1.42(1) 1
C2 O1	1.36(1) 1	C16 O5	1.42(1) 1	C24 C25	1.42(2)
C3 H3	0.93 1	C17 H17A	0.96 1	C24 O13	1.35(1) 1
C3 C4	1.41(1)	C17 H17B	0.96 1	C25 C26	1.43(2)
C4 H4	0.93 1	C17 H17C	0.96 1	C25 C28	1.43(2)
C4 C5	1.36(1)	C17 O6	1.38(2) 1	C26 C27	1.38(2)
C5 C6	1.42(2)	C18 H18B	0.96 1	C26 O11	1.36(1) 1
C5 C7	1.49(2)	C18 H18C	0.96 1	C27 O10	1.37(1) 1
C6 H6	0.93 1	C18 O7	1.38(1) 1	C28 C29	1.40(2)
C7 C8	1.35(2)	O1 H1A	0.821 1	C28 O12	1.27(1) 1
C7 O2	1.37(1) 1	O3 H3A	0.820 1	C29 H29	0.93 1
C8 H8	0.93 1	O4 H4A	0.819 1	C29 C30	1.34(1)
C8 C9	1.41(2)	C19 H19A	0.96 1	C30 C31	1.45(1)
C9 C10	1.47(2)	C19 H19B	0.96 1	C30 O13	1.38(1) 1
C9 O3	1.28(1) 1	C19 H19C	0.96 1	C31 C32	1.43(1)
C10 C11	1.37(1)	C19 O10	1.35(1) 1	C31 C36	1.36(2)
C10 C15	1.40(2)	H20A	0.96 1	C32 H32	0.93 1
C11 C12	1.40(2)	H20B	0.96 1	C32 C33	1.34(1)
C11 O2	1.38(1) 1	H20C	0.96 1	C33 H33	0.931 1
C12 C13	1.37(2)	C20 O8	1.41(1) 1	C33 C34	1.37(1)
C12 O7	1.34(1) 1	C21 H21A	0.96 1	C34 C35	1.38(2)
C13 C14	1.39(2)	C21 H21C	0.96 1	C34 O14	1.37(1) 1
C13 O6	1.39(1) 1	C21 H21B	0.96 1	C35 H35	0.93 1
C14 C15	1.37(2)	C21 O9	1.45(1) 1	C35 C36	1.37(2)
C14 O5	1.38(1) 1	C22 C23	1.40(2)	C36 H36	0.93 1
O11 H11	0.820 1	O12 H12	0.822 1	O14 H14	0.823 1

The crystal data and structure refinement for xanthomicrol (**1**) is summarized in Table 4.

**Table 4:** Crystal data and structure refinement for xanthomicrol (**1**).

Parameter	Value
Empirical formula	C_18_H_16_O_7_
*M*	344
Crystal system	Orthorhombic
Space group	*Pna21*
a (Å)	18.8736(14)
b (Å)	4.1108(3)
c (Å)	40.448(3)
α (°)	90.00
β (°)	90.00
γ (°)	90.00
Volume (Å^3^)	3138.2(4)
Z	8
D_c_/g·cm^−3^	1.389
Absorption Coefficient (Mo Kα) mm^−1^	0.111
Crystal size (mm)	0.04 × 0.10 × 0.45
Theta range for data collection (θ Range/°)	2.014° to 25.344°
Index Ranges	−15 ≤ h ≤ 15, −3 ≤ k ≤ 3, −32 ≤ l ≤ 31
Reflections collected	3203
Unique reflections, R_int_	1518 [R(int) = 0.0512]
Completeness of theta = 25.50°	92%
Refinement Method	Full-matrix least-squares on F^2^
Data/restraints/parameters	3203/1/463
Goodness of fit on F^2^	1.030
Final R indices [I > 2sigma(I)]	R1 = 0.1111, wR2 = 0.0584 I > 2σ(I)
R indices	R1 = 0.1460, wR2 = 0.1314
Largest Diff. peak and hole (e·Å^−3^)	0.210 and −0.198 e·Å^−3^

## 3. Experimental Section

### 3.1. Isolation of Flavonoids

Compounds **1**–**3** and **4** were previously isolated from aerial parts of *S. stricta* [13] and *S. lycia* [2], and the structures of the compounds were established by spectroscopic evidence (UV, IR, MS, NMR). Before the activity studies, structure elucidation of compounds **1**–**4** was reconfirmed by spectral (UV, MS and ^1^H-NMR) analysis [2,13,14,16,44].

### 3.2. Screening of hMAO Inhibitory Activities

#### 3.2.1. Chemicals

hMAO-A and hMAO-B (both recombinant, expressed in baculovirus-infected BTI insect cells), *R*-(–)-deprenyl hydrochloride (selegiline), moclobemide, resorufin, dimethyl sulfoxide (DMSO), and other chemicals were purchased from Sigma-Aldrich (Munich, Germany). The Amplex^®^-Red MAO assay kit (Cell Technology Inc., Mountain View, CA, USA) contained benzylamine, *p*-tyramine, clorgyline, pargyline and horseradish peroxidase.

#### 3.2.2. Determination of hMAO-A and hMAO-B Activities

The activities of hMAO-A and hMAO-B were determined using p-tyramine as common substrate and calculated as 161.50 ± 8.33 pmol/mg/min (*n* = 3) and 140.00 ± 7.99 pmol/mg/min (*n* = 3), respectively. The interactions of the synthesized compounds with hMAO isoforms were determined by a fluorimetric method described and modified previously [45,46]. The production of H_2_O_2_ catalyzed by MAO isoforms was detected using Amplex^®^-Red reagent, a non-fluorescent probe which reacts with H_2_O_2_ in the presence of horse radish peroxidase to produce the fluorescent product resorufin. The reaction was started by adding 200 µM Amplex Red reagent, 1 U/mL horseradish peroxidase (HRP), and *p*-tyramine (concentration range 0.05–0.5 mM). Control experiments were carried out by replacing the compound and reference inhibitors. The possible capacity of compounds to modify the fluorescence generated in the reaction mixture due to non enzymatic inhibition was determined by adding these compounds to solutions containing only the Amplex Red reagent in a sodium phosphate buffer. No modification was recorded. The possible capacity of compounds to inhibit HRP in the test solution was also determined. The compounds did not inhibit HRP in the experimental medium.

#### 3.2.3. Kinetic Experiments

Synthesized compounds were dissolved in dimethyl sulfoxide, with a maximum concentration of 1% and used in the concentration range of 1.00–100.00 µM for compounds **1** and **4**; 5.00–100.00 mM for compounds **2** and **3**. Kinetic data corresponding the interaction of hMAO isoforms with the compounds were determined using computer-fit calculation (Prism 4.0, GraphPad SoftwareThe slopes of the Lineweavere-Burk plots were plotted *versus* the inhibitor concentration and the K_i_ values were also determined from the x-axis intercept as -K_i_. Each K_i_ value is the representative of single determination where the correlation coefficient (R2) of the replot of the slopes *versus* the inhibitor concentrations was at least 0.98. SI was calculated as K_i_(hMAO-A)/K_i_(hMAO-B. The protein was determined according to the Bradford method [47].

#### 3.2.4. Reversibility Experiments

Reversibility of the MAO inhibition with newly synthesized compounds was determined by centrifugation-ultrafiltration method previously described [48]. Recombinant enzymes (hMAO-A or B) were incubated with a single concentration of the synthesized compounds or the reference inhibitors in a sodium phosphate buffer (0.05 M, pH 7.4) for one hour at 37 °C. An aliquot was stored at 4 °C and used for the measurement of MAO-A and -B activities. The remaining incubated sample was placed in an Ultrafree-0.5 centrifugal tube with a 30 kDa Biomax membrane and centrifuged at 9000× *g* for 20 min at 4 °C. The enzyme retained in the 30 kDa membrane was re-suspended in a sodium phosphate buffer at 4 °C and centrifuged two more times. After the third centrifugation, the enzyme retained in the membrane was re-suspended in sodium phosphate buffer (300 mL) and an aliquot of this suspension was used for MAO-A and -B activity determination. Control experiments were performed simultaneously to determine the 100% MAO activity by replacing the compounds and standards with appropriate dilutions of the vehicles. The corresponding values of percent (%) MAO isoform inhibition was separately calculated for samples with and without repeated washing.

#### 3.2.5. Molecular Docking Studies

Molecular models of the inhibitors (**1**,**4**) were built and optimized using SPARTAN 10.0 at PM3 level on iMac 3,4 GHz Intel Core i7 processor. The crystal structures of MAO-A, and MAO-B were extracted from the Protein Data Bank (PDB) [49] Their PDB codes are 2Z5X and 2V5Z for MAO-A and MAO-B respectively. Each structure was cleaned of all water molecules and inhibitors as well as all non-interacting ions were removed before being used in the docking studies. For MAO-A and MAO-B, one of the two subunits was taken as the target structure. For each protein all hydrogens were added and minimized using “Clean Geometry” toolkit, then for more complete optimization final geometry was submitted to “Prepare Macromolecule” protocol of Discovery Studio (Accelerys) assigning CHARMM force field. Missing hydrogen atoms were added based on the protonation state of the titratable residues at a pH of 7.4. Ionic strength was set to 0.145 and the dielectric constant was set to 10. Autodock Tool (ADT) interface was used for the preparation of input files of macromolecules and ligands for docking calculations. Docking was achieved using AutoDock 4.2.6 coding scripts [50] using HPxw8600-Work-Station. The detailed docking procedures are given elsewhere [51,52]. Acelrys Visualization 4.0 program was used for rendering the 2D and 3D pictures. To render visible the detailed interactions of the docked poses of the inhibitors (**1**,**4**) Acelrys Visualization 4.0 program was used.

### 3.3. X-ray Analysis

Single crystal X-ray diffraction patterns of xanthomicrol were collected at 293 K with Bruker APEX-II CCD diffractometer from a K780 X-ray generator applying 50 kV and 40 mA. All data were accumulated using a graphite monochromated Mo *K*α (λ = 0.71073 Å) radiation. The least-squares methods was applied on the basis of all reflections with *F*^2^ > 2σ(*F*^2^) to determine the lattice parameters.

## 4. Conclusions 

The present study provides an evidence for the isolated flavonoids from *Sideritis* species as new potent and selective hMAO-inhibitors. The results suggest two compounds, xanthomicrol and salvigenin as potential natural starting molecules for developing novel selective MAO-A inhibitors, for prevention and treatment of psychiatric disorders such as depression and anxiety, and also cognitive impairments in Alzheimer’s and Parkinson’s Diseases. Current study gives a clear insight in the inhibitory ability of these flavonoids towards hMAO; however, more studies are needed to clarify their efficacy *in vivo*. In addition, in the present study, crystal structure of xanthomicrol, which is the most selective MAO-A inhibitor tested, has been introduced for the first time.

## Supplementary Data

Additional material available from Cambridge Crystallographic Data Center as Deposition No. CCDC 1051489 comprises H-atom coordinates, thermal parameters and remaining bond lengths and angles. These data can be obtained free of charge via http://www.ccdc.cam.ac.uk/conts/retrieving.html, or from the Cambridge Crystallographic Data Centre, 12 Union Road, Cambridge CB2 1EZ, UK; E-Mail: deposit@ccdc.cam.ac.uk.
